# Neutronics design of shutdown and control systems for a Zero Power Experiments of chloride-based molten salt fast reactor

**DOI:** 10.1371/journal.pone.0309928

**Published:** 2024-10-16

**Authors:** Lakshay Jain, Omid Noori-kalkhoran, Lewis Powell, Andrew Jones, Daliya Aflyatunova, Bruno Merk

**Affiliations:** 1 School of Engineering, University of Liverpool, Liverpool, United Kingdom; 2 School of Engineering, Cardiff University, The Parade, Wales, United Kingdom; 3 School of Physical Sciences, The University of Liverpool, Liverpool, United Kingdom; Khalifa University of Science and Technology, UNITED ARAB EMIRATES

## Abstract

Nuclear power’s role as a reliable, baseload, low-carbon source and its importance in achieving clean energy goals are being increasingly recognized with growing urgency around decarbonization of the global energy systems. However, to deliver a long-term sustainable solution, it is essential to develop innovative nuclear technologies for improving the fuel utilization and reducing the nuclear waste disposal challenge. Zero Power Reactors (ZPR) are an essential initial step for developing new nuclear technologies because they allow for testing and refinement in a safe environment before large-scale deployment. This paper discusses the design of a ZPR experiments for the development of iMAGINE, a novel chloride-based molten salt reactor technology. The paper presents a detailed analysis of the neutronic design for the shutdown and control systems of an experimental ZPR based on the iMAGINE molten salt reactor technology. The study concludes that a split-core design with a lower corner reflector as an extension of the lower annular reflector offers the most robust ZPR configuration, offering optimum operational margins and maneuverability. This design ensures safety, regulatory compliance, and sufficient control and shutdown performance for the successful development of the iMAGINE technology.

## 1. Introduction

The United Nations has defined the challenge for all future energy systems through Sustainable Development Goal 7 (UN SDG 7)–ensuring access to affordable, reliable, sustainable, and modern energy for all [1]. With the growing climate change emergency and the need for decarbonization of global energy systems, the importance of nuclear power in achieving net-zero targets is being increasingly recognized across the world [2–5]. It is currently the only freely manageable, very low-carbon energy technology with 24/7 availability to complement intermittent renewables [6]. Consequently, more than 20 nations recently announced the goal of tripling global nuclear energy capacity by 2050 during the World Climate Action Summit of COP28 [7].

Although nuclear energy has undoubted potential as a source of baseload energy while reducing CO_2_ emissions [5], there is a strong need for the development of advanced nuclear technologies for improvement in sustainability, economics, safety, reliability and proliferation-resistance for long-term success and delivering on future requirements [8, 9]. iMAGINE, an innovative nuclear system based on chloride molten salt fast reactor technology, was proposed by Merk et al. to deliver on the aforementioned principles [10, 11]. It has been optimized for integrated closed fuel cycle operation for improved fuel utilization as well as solving the nuclear waste disposal problem. A comprehensive four-step plan–basic studies, experimental zero power reactor, small-scale demonstrator, and industrial demonstrator–has been envisaged for the development of iMAGINE to ensure active risk reduction and quick progress [11, 12]. The importance of such a stepwise development paradigm for a game-changing technology like iMAGINE has been previously highlighted [11, 13, 14] and coincides well with the observations of General L. Groves, Project Lead, Manhattan Project about chances and risk taking [15].

This paper focuses on the neutronic design of an experimental zero power reactor (ZPR) for the iMAGINE system. A zero power experimental facility is a crucial initial step for the development of a novel nuclear technology because of the following advantages [6]:

1. Lower costs and risks as compared to a large-scale project or demonstrator;
2. Faster development timelines (< 5 years);
3. Ability to train experts in the design, construction, and operation of future nuclear facilities in a low-complexity setting;
4. Possibility to prove the quality and reliability of design tools;
5. Possibility for design and testing of novel instrumentation and systems; and
6. Mutual opportunity for developers and regulators to develop the regulatory framework for non-light water reactor technologies.

Overall, zero power facilities provide a safe environment to test and refine innovative nuclear technologies before large-scale deployment and offer the following broad benefits [6]:

1. Supporting the entire reactor development process (design, licensing, construction, commissioning, and operation);
2. Training a new generation of skilled nuclear workforce; and
3. Establishing scientific leadership in advanced reactor technologies

The present work aims to investigate and consolidate the ideas presented in Ref. [6] with rigorous modeling and simulation results, as appropriate at the current design stage. Comprehensive analyses of the neutronic design of shutdown and control systems for a Zero Power Experiment (ZPE) as an important stepping stone towards the development of iMAGINE, and more generally, chloride-based molten salt fast reactors have been presented in this paper. The analysis is based on the steady state neutron transport equation (no time dependence), and thus, neutron precursor concentrations don’t affect the system. The ZPR is assumed to operate at room temperature (without any external heating) due to which the fuel is solid with no flow. However, since steady state conditions are considered, neither flow nor precursor concentrations (thus, delayed neutrons) will affect the system.

It must also be mentioned here that a two-step operation is planned for the proposed experimental ZPR—first with fuel salt in the solid phase at room temperature followed by molten fuel salt at elevated temperatures—in close collaboration with the nuclear regulator. The solid fuel in the first step will provide higher safety and reduced complexity to characterise the system and perform essential reactor physics experiments, like critical experiments, neutron flux measurements, etc. These experiments can then be used to improve, validate, and qualify the calculation models as well as codes. This step will also involve the gathering of the necessary nuclear data. Subsequently, the confidence gained from a well characterized solid salt based ZPR and validated models will be used for step two with molten fuel, most likely through external heating. This stepwise approach has been planned for active risk mitigation and high engagement with the regulator at each step. Such an approach will enable mutual learning and quick development of this promising technology, as well as developing the necessary regulatory framework. After the initial reactor physics experiments and required characterization of the system, the proposed experimental ZPR can also be used to perform experiments with different salts leading to a versatile multi-purpose test facility. Additionally, we are also actively researching advanced experimentation and instrumentation technologies for testing a variety of salts in a given neutron field. The manuscript is organized as follows: Section 2 describes the geometrical and material configuration for the basic ZPE setup. This is followed by a detailed discussion about the shutdown and control systems, and evaluation of their respective worth in Section 3. Design of the selected ZPE system is summarized in Section 4 and Finally, the conclusions have been presented in Section 5.

## 2. Codes, model and methods

**Fig 1:**
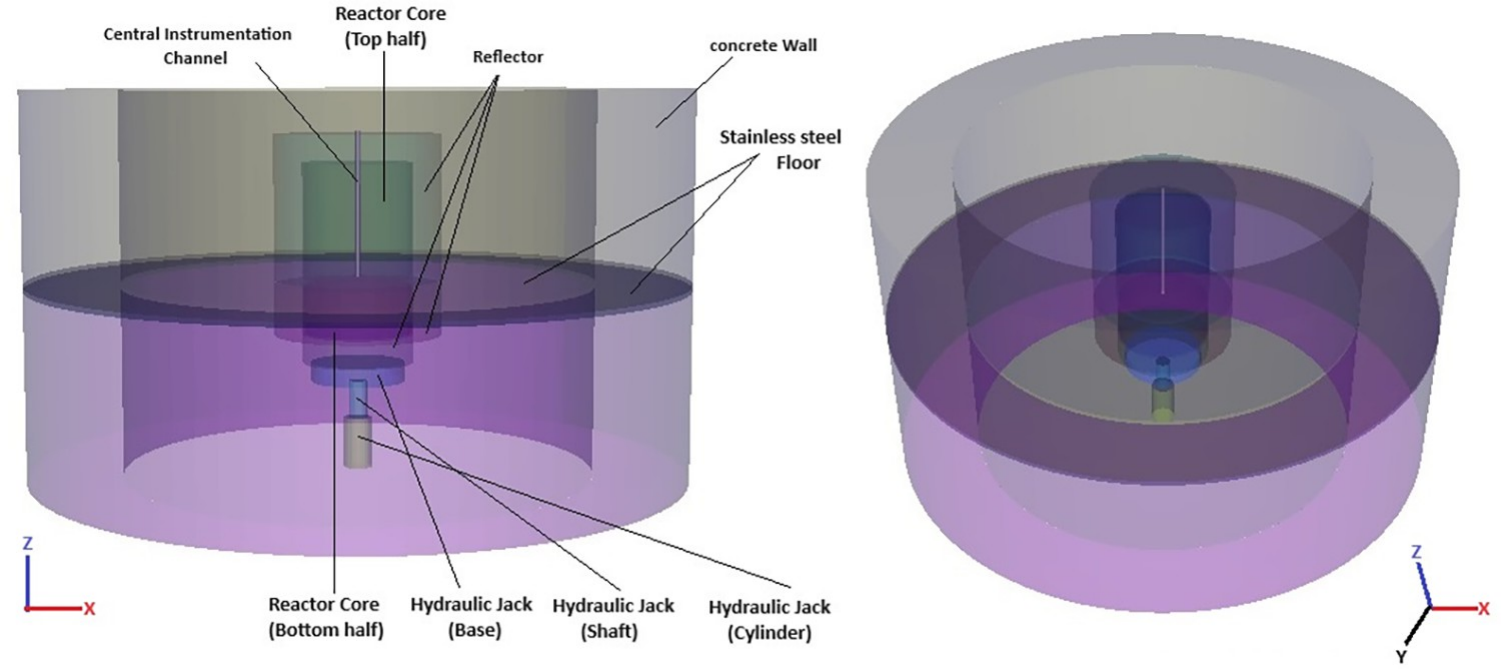
3D illustration of the experimental zero power reactor (ZPR) system.

**Fig 2:**
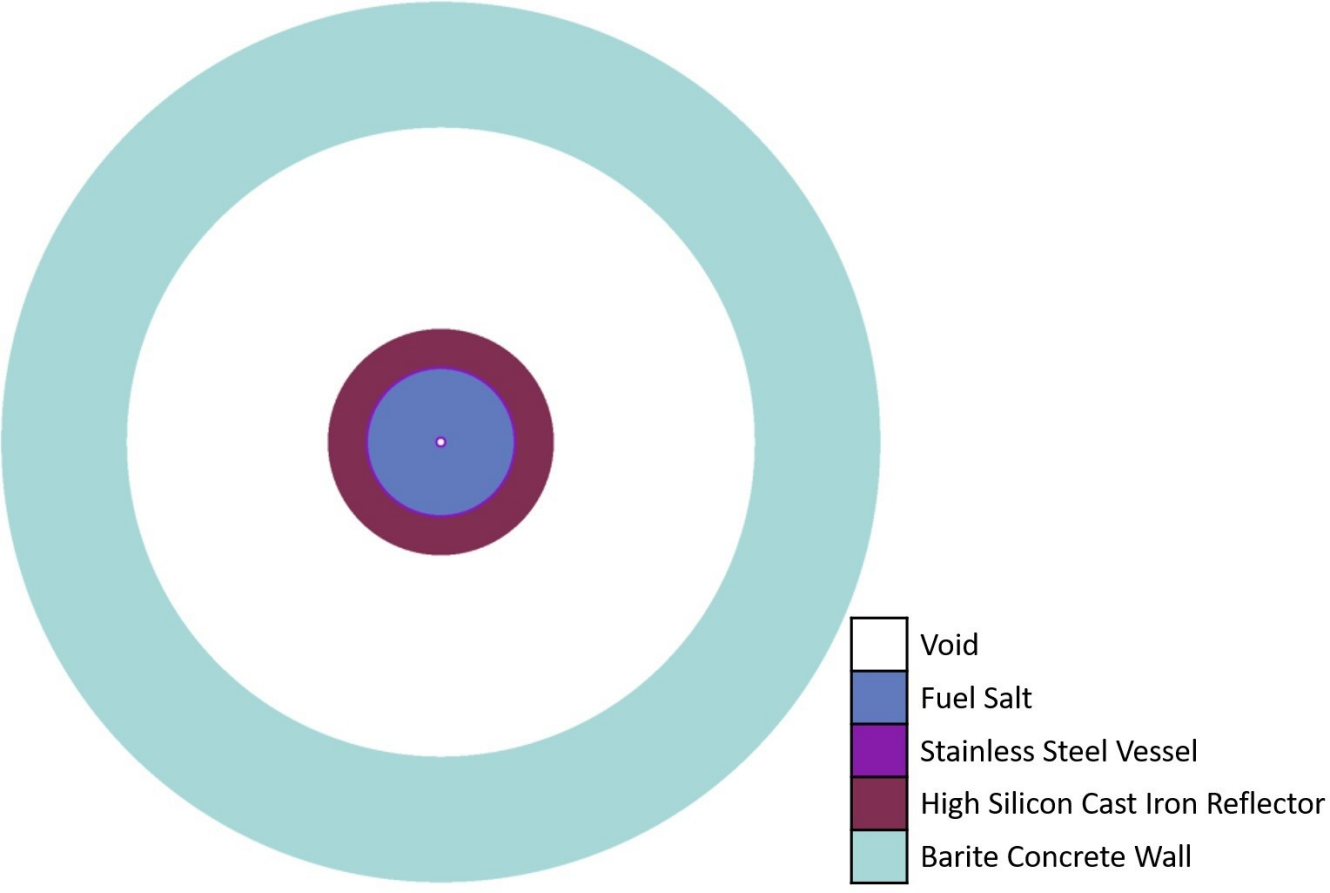
Radial (XY) slice of upper part of the zero power experimental (ZPE) system model at an axial distance of 10 cm from the stainless steel floor.

**Fig 3:**
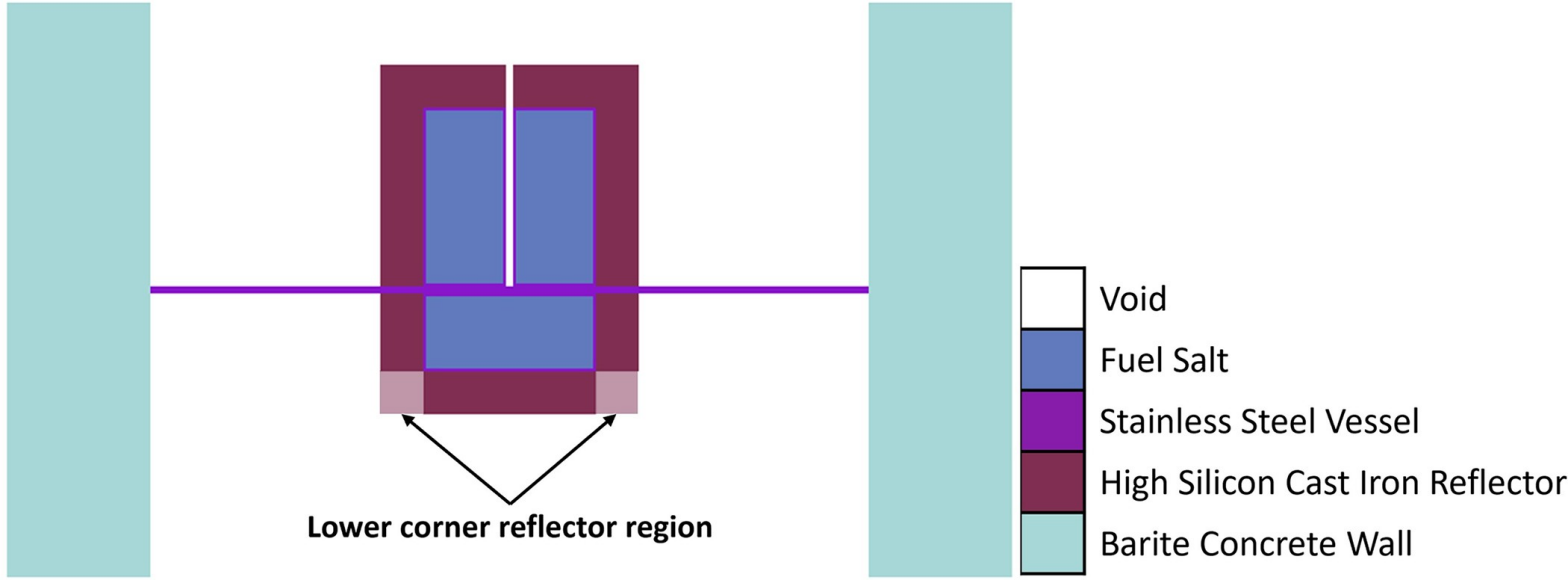
Central axial (XZ) cross-sectional view of the ZPE system model.

A split-core design has been proposed for the ZPE system where the experimental and operational parts of the reactor are split from each other and placed in two physically separated areas (see Figs 1–3). This novel and innovative design originated through a series of workshops held in Fall 2023 within the Reactor Physics Group, University of Liverpool. It has been proposed primarily because of the following reasons [6]:

1. It eliminates the possibility of inadvertent addition of positive reactivity within the system in the accidental scenario of failure of the upper core anchoring during system shutdown leading to the upper part falling onto the lower core; and
2. Nuclear regulators are increasingly inclined towards the separation of operational and experimental teams, as recently highlighted during the NEA workshop on ‘*The demise of zero power reactors*’ [16]. The proposed design takes this requirement forward, embeds it into the design and construction of the system, and thus, ensures complete independence of the reactor operation from experiments. Consequently, ease of access to the experimental hall, for both personnel and equipment, can be ensured while still maintaining strict access-control to the operational areas limiting the possibility of misuse through any unauthorized third-party access; and
3. It provides the possibility of a simple and effective shutdown mechanism by moving the two parts away from each other. It can be swiftly actuated in the case of an emergency, promptly providing a large negative reactivity insertion. The shutdown mechanism is also fail-safe and ensures passive safety (gravity assisted) within the system by moving away the lower part while the upper part of the core rests on the intermediate floor (see Fig 3) and is stationary.

An exhaustive discussion of the design ideology can be found elsewhere (see Ref. [6]). Fig 2 shows a radial slice of the experimental (upper) part of the computational model at an axial distance of 10 cm from the stainless steel floor. It also represents the reference 2D computational model investigated in this paper. A view of the central axial cross-section of the ZPE computational model has been given in Fig 3.

The fuel composition for the ZPE is based on the chloride salt system chosen for iMAGINE. It consists of NaCl-UCl_3_-UCl_4_ in the eutectic composition of 42.5%– 17.0%– 40.5% (with 35% U-235 and 99.9% Cl-37 enrichment). It must be noted here that the 35% U-235 enrichment is the current working hypothesis but could be changed based on the requirements of the country where such a facility is located. Similarly, 99.9% Cl-37 enrichment is also a working hypothesis which must be optimized based on the enrichment cost, criticality loss and, later, breeding demand for a full reactor. A detailed discussion on the data of the salt system and the rationale behind the choice is given in Ref. [17]. The solid fuel salt has a radius and total height of 58 cm and 170 cm, respectively. The initial core dimensions for the reference model were derived through a parametric study which is presented in Section 3.1.1. The fuel is contained in a stainless steel (SS304) vessel of thickness 2 cm surrounded by a high-silicon cast iron reflector (Hi-Si Cast Iron) [18] of thickness 30 cm in both axial and radial directions. However, as seen in Fig 3, the reflector is present in the upper corner of the ZPE system but absent in the lower corner (Upper corner of the system refers to the location where the upper axial and upper annular reflectors intersect each other and Lower corner of the system refers to the location where the lower axial and lower annular reflectors intersect each other). This configuration was chosen as the presence of upper corner reflector reduces heterogeneity within the experimental part, while absence of reflector material in the lower corner could potentially provide better operational performance. The effect of lower corner reflector on the operational characteristics of the ZPE system will be investigated in Section 3.2, Section 3.3, and Section 3.5. As mentioned earlier, the experimental (upper) and operational (lower) parts of the ZPE are separated using an intermediate, 5 cm thick stainless steel (SS304) floor. It must be noted here that the thickness of the intermediate stainless steel floor is a matter of optimization and discussion with the regulator. A central experimental channel of radius 2.5 cm is also modeled in the upper core to closely represent a realistic ZPE setup. It must be noted that the actual ZPE will consist of additional vertical as well as horizontal experimental channels [6]. However, these have not been considered here as the simplified model used currently is sufficient for the design studies presented in this paper. The entire ZPE assembly is surrounded by a 1 m thick and 4 m high biological shield of barite concrete. For simplification, no biological shield has been considered in the axial direction and vacuum boundary conditions were applied on all sides. Detailed geometrical parameters for the system have been shown in Table 1. Since ZPE systems are designed to operate at extremely low-power levels (< ~10 kW) to eliminate the need for any heat removal systems as well as ensure very low radiation levels, all materials are considered to be at room temperature of 27 °C (300 K). The detailed material configuration and compositions have been provided in Tables 2 and 3, respectively.

**Table 1:** Geometrical dimensions of the reference Zero Power Experiment (ZPE) system.

Parameter	Dimensions
***Upper Core***	
Fuel height [cm]	120
Fuel radius [cm]	58
Cladding thickness [cm]	2
Annular reflector thickness [cm]	30
Axial reflector height [cm]	30
Experimental channel radius [cm]	2.5
***Lower Core***	
Fuel height [cm]	50
Fuel radius [cm]	58
Cladding thickness [cm]	2
Annular reflector thickness [cm]	30
Axial reflector height [cm]	30
***Other structures***	
Floor plate [cm^3^]	500 × 500 × 5
Wall inner radius [cm]	250
Wall thickness [cm]	100
Wall height [cm]	400

**Table 2:** Material configuration of the Zero Power Experiment (ZPE) system.

Parameter	Material	Density [g/cm^3^]
***Upper Core***		
Fuel salt	NaCl-UCl_3_-UCl_4_ eutectic	3.2112
Vessel/Fuel clad	Stainless steel (SS304)	7.94
Annular reflector	High silicon cast iron	7.01
Axial reflector	High silicon cast iron	7.01
***Lower Core***		
Fuel salt	NaCl-UCl_3_-UCl_4_ eutectic	3.2112
Vessel/Fuel clad	Stainless steel (SS304)	7.94
Annular reflector	High silicon cast iron	7.01
Axial reflector	High silicon cast iron	7.01
***Other structures***		
Floor plate	Stainless steel (SS304)	7.94
Wall	Barite concrete	3.35

**Table 3:** Material compositions.

Material	Atomic/Weight Fraction [%]
***NaCl-UCl***_***3***_***-UCl***_***4***_ ***eutectic*** ^†^	
NaCl	42.5
UCl_3_	17.0
UCl_4_	40.5
***Stainless steel (SS304)*** ^‡^	
C	0.080
Si	1.000
P	0.045
Cr	19.000
Mn	2.000
Fe	68.375
Ni	9.500
***High silicon cast iron*** ^‡^ [18]	
C	0.500
S	0.014
Si	15.320
Fe	84.166
***Barite concrete*** ^‡^ [19]	
H	0.3585
O	31.1622
Mg	0.1195
Al	0.4183
Si	1.0457
S	10.7858
Ca	5.0194
Fe	4.7505
Ba	46.3400

† –atomic fraction

‡ –weight fraction

The simulations have been performed with OpenMC [20] using 10^6^ particles with a total of 100 batches (10 inactive and 90 active batches) and are based on ENDF/B-VII.1 nuclear data library [21]. These detailed continuous energy Monte-Carlo calculations lead to a standard deviation of ~8 pcm which is appropriate for this type of neutronics analyses. Results of the reference case, both 2D as well as 3D, have been benchmarked against other reputable monte carlo codes such as MCNPX-2.7 [22], Keno-VI [23] from the SCALE-6.2.3 package [24] and HELIOS-v2.03 [25] to check the validity and convergency and presented in Section 3.1.

## 3. Results and discussion

This section focuses on the neutronics design studies for the proposed ZPE system. It should be noted here that although the current analyses are aimed towards chloride-based molten-salt fast reactors, the design philosophy and methodology used in this paper would also be applicable for the neutronics design of the control and shutdown systems of other molten salt reactors, irrespective of the salt type. The present section is organized as follows–firstly, 2D and 3D calculation results for the reference model using OpenMC along with their benchmarking against other Monte-Carlo codes have been given in Section 3.1. This is important since chloride based molten salt fast reactors could have neutron energy spectrum which is very different from other reactor types and a significant fraction of neutrons have energies in the unresolved resonance region [26]. Next, different possibilities for the shutdown and control systems have been discussed in Section 3.2 and Section 3.3, respectively. This is required because multiple combinations of the shutdown and control systems are possible, and it is essential to evaluate the operational envelope they offer to select the most optimum configuration. The analyses of shutdown and control systems are followed by investigation of the sizes of the experimental and operational parts for the split-core design in Section 3.4 to help optimize the dimensions of the two parts. Finally, the net worth of the chosen shutdown and control systems in the envisaged operational state of the ZPE has been studied in Section 3.5. It must be mentioned here that the entire optimization process is non-linear and, thus, the sequence of scoping studies used here might not reflect the real process.

### 3.1 Reference model

The proposed ZPE system is modelled using OpenMC and compared with results from MCNPX-2.7 and Keno-VI for cross-validation (Keno-VI calculations have been performed in the continuous energy as well as multi-group mode with 252 energy groups). The geometrical (see Figs 2 and 3) and material (see Tables 1–3) configurations have been given earlier in Section 2. The results of this cross-validation and benchmarking exercise for the 3D case have been presented in Table 4. The results show excellent agreement with a maximum difference of 95.21 ± 15.57 pcm between OpenMC and MCNPX-2.7. Although the results from different codes are very close, they must be validated against actual experiments as unlike other reactors, a significantly large fraction of the neutrons have energies in the unresolved resonance region. No theoretical models exist for unresolved resonance treatment and all codes, stochastic or deterministic, rely on p-tables which are adjusted to match experimental results. Thus, the cross-validation exercise performed here should not be interpreted as doing away with the need for experimental validation and is only to understand whether the subsequent analyses can be based on a single code.

**Table 4:** Benchmarking results for reference ZPE model in 3D.

Code	Multiplication Factor, k	Difference [pcm]
OpenMC	1.01623 ± 0.00008	-
MCNPX-2.7	1.01725 ± 0.00014	95.21 ± 15.57
Keno-VI	1.01661 ± 0.00009	36.69 ± 11.44

Similarly, the results for 2D case of the reference computational model have been presented in Table 5. Like the 3D reference model, results from different codes for the 2D case are also in excellent agreement with each other with a maximum difference of 166.94 ± 6.09 pcm between OpenMC and HELIOS. However, it is interesting to see a difference of almost 970 pcm between the multiplication factors obtained from HELIOS using its 173 and 316 energy group libraries. This is because the experimental ZPR based on the iMAGINE system has a significantly harder neutron spectrum [26] and a coarse energy group structure for fast neutrons can lead to large discretization errors. This clearly shows the need to use a sufficient number of fast energy groups for multi-group neutronic calculations.

**Table 5:** Benchmarking results for reference ZPE model in 2D.

Code	Multiplication Factor, k	Difference [pcm]
OpenMC	1.14908 ± 0.00008	-
Keno-VI	1.14959 ± 0.00007	38.61 ± 8.05
HELIOS (173g) ^†^	1.159803	804.83 ± 6.09
HELIOS (316g) ^‡^	1.146877	-166.94 ± 6.09

† –using 173 energy group library of HELIOS, CCCP solver

‡ –using 316 energy group library of HELIOS, CCCP solver

#### 3.1.1 Initial core dimensions

As mentioned earlier, optimizing the overall ZPR system is a non-linear process which begins with some initial estimate for the system dimensions (iTeration process). These “seed” values are based on the experience and expertise of the design team. However, these initial dimensions are not fixed and undergo a parametric investigation to find appropriate values (used earlier in the reference model). The results of these parametric calculations have been shown in Table 6, where the total core height and radius are varied from 160 cm to 180 cm and 50 cm to 65 cm, respectively. The aim is to identify those core dimensions for which the system has an excess reactivity between 1000 pcm and 2000 pcm. This is sufficient to allow operational maneuverability during various experiments but not too high that it is difficult to control. It is also desirable to have a core diameter to height ratio of around 2:3 for the overall system. This is because a split-core design has been chosen for the ZPE system and it would be ideal to have a core height to diameter ratio of unity in the upper (experimental) part for maximum homogeneity. Based on these criteria, core height and radius of 170 cm and 58 cm, respectively, are found to be most suitable. It is evident that for most cases considered in Table 6, either the system lacks sufficient excess reactivity required for experiments or has too much excess reactivity. Only two other cases–core height and radius of 160 cm and 60 cm, as well as 180 cm and 58 cm, respectively–are close enough to the abovementioned criteria. However, the ratio of core height to diameter is further away from the desired value for the former, while the system has an excess reactivity of around 2400 pcm for the latter. Thus, the initial dimensions for the reference model have been chosen as 170 cm and 58 cm of core height and radius, respectively.

**Table 6:** Multiplication factor of ZPR for different core dimensions.

Core Height [cm]	Core Radius [cm]	Multiplication Factor, k
160	50	0.93972 ± 0.00007
160	56	0.99180 ± 0.00008
160	58	1.00752 ± 0.00008
160	60	1.02265 ± 0.00008
160	65	1.05742 ± 0.00007
170	50	0.94774 ± 0.00007
170	56	1.00029 ± 0.00008
**170**	**58**	**1.01623 ± 0.00008**
170	60	1.03137 ± 0.00008
170	65	1.06642 ± 0.00009
180	50	0.95503 ± 0.00009
180	56	1.00797 ± 0.00009
180	58	1.02408 ± 0.00008
180	60	1.03926 ± 0.00008
180	65	1.07495 ± 0.00008

### 3.2 Shutdown system

**Fig 4:**
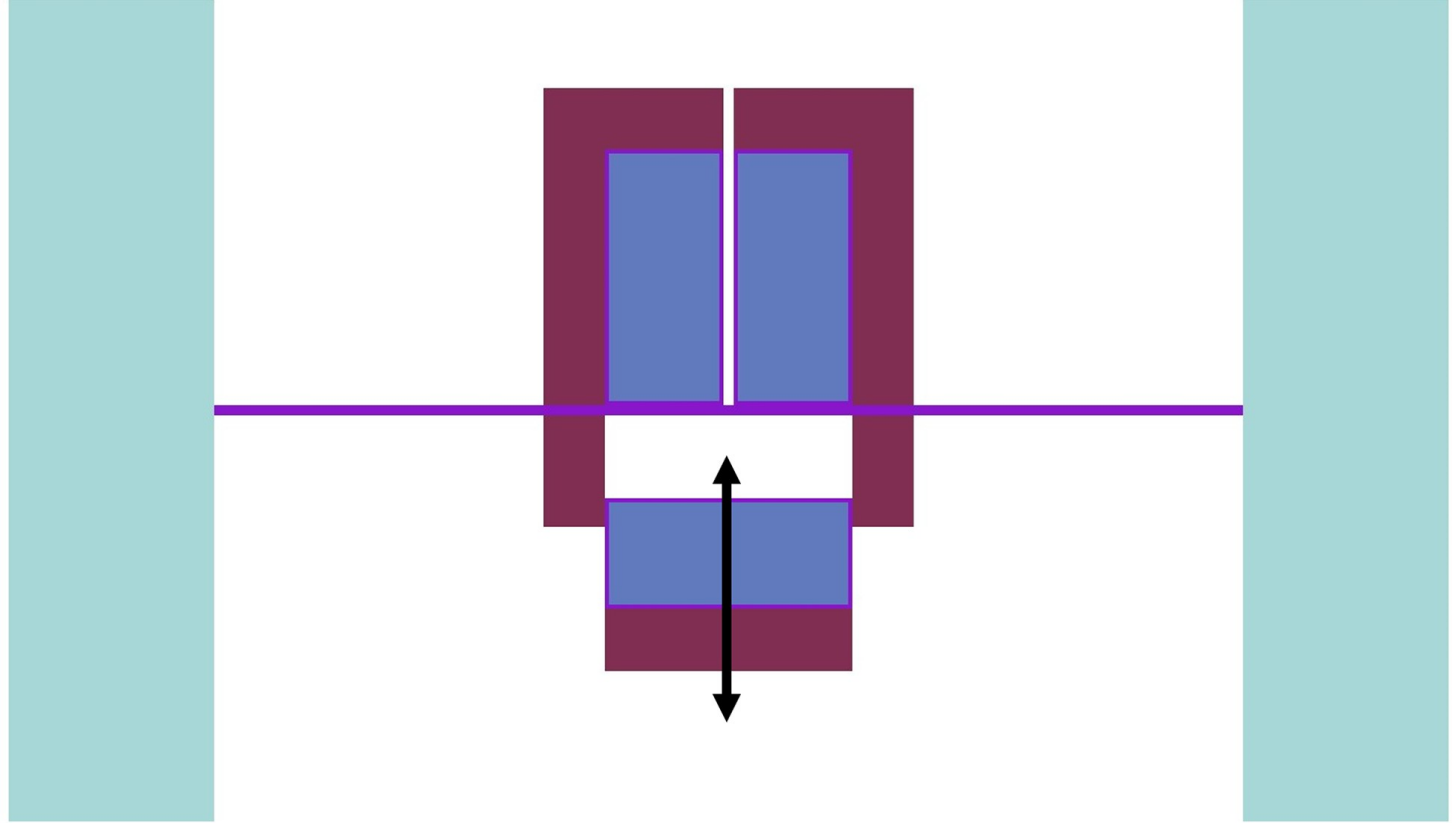
Shutdown using core split.

**Fig 5:**
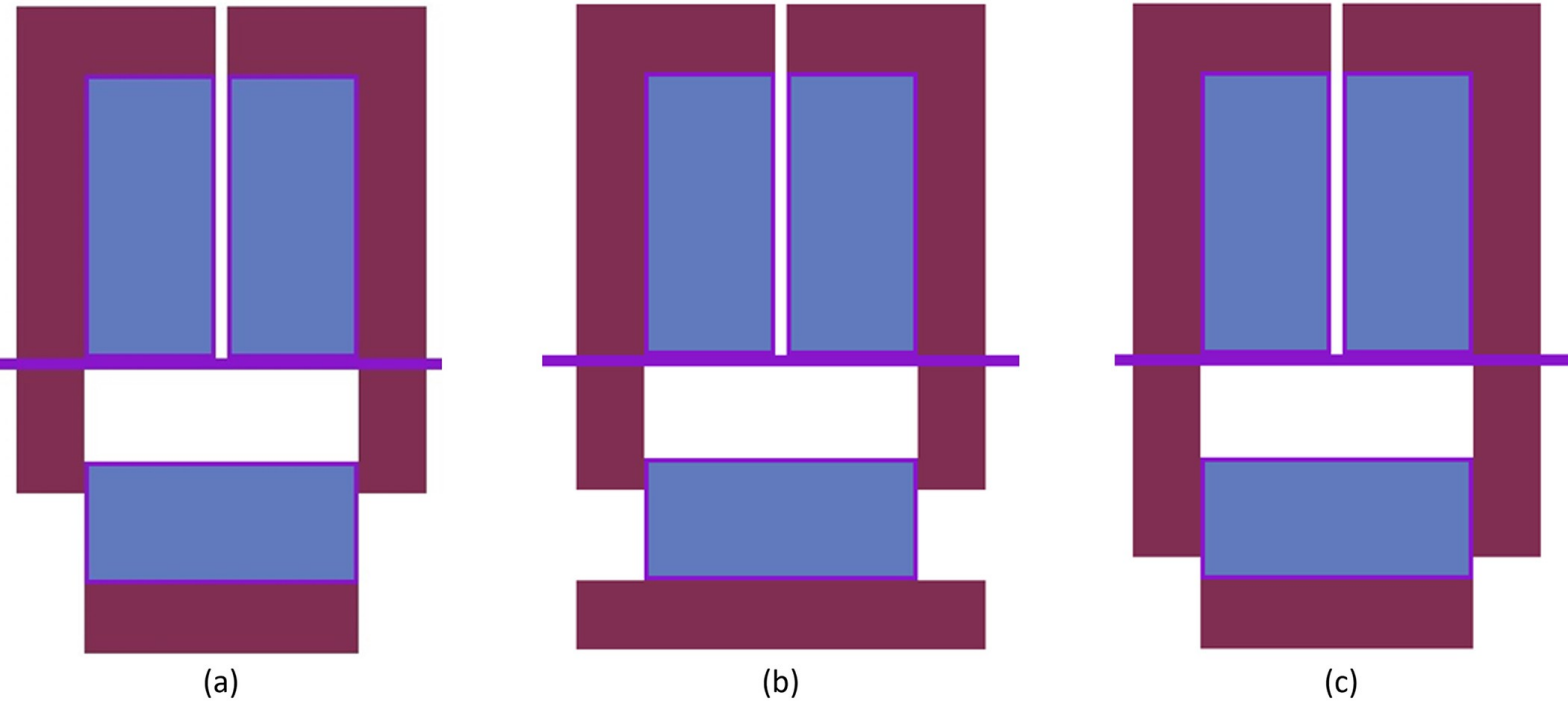
ZPE assembly configuration (a) without lower corner reflector; (b) with lower corner reflector as lower axial reflector; (c) with lower corner reflector as lower annular reflector.

The ZPE system has been designed in a split-core configuration with a stationary upper part for experimentation and a movable lower portion for reactor operations and safe shutdown. The reasons for choosing such a novel design have been outlined earlier in Section 2 and a detailed discussion can be found elsewhere (see Ref. [6]). The upper part of the split-core configuration rests on an intermediate stainless steel floor while the lower core (along with the lower axial reflector) can be moved vertically for shutdown, as shown in Fig 4. The ZPE assembly could be designed with or without the presence of reflector material at the lower corner of the system (see Fig 3). This would lead to three different configurations as follows:

1. ZPE without lower corner reflector, as considered in the reference model (see Fig 5A)
2. ZPE with lower corner reflector as an extension of the lower axial reflector (see Fig 5B)
3. ZPE with lower corner reflector as an extension of the lower annular reflector (see Fig 5C)

All three cases are modeled using OpenMC with the calculation settings given in Section 2. The aim is to investigate the total shutdown worth as well as the rate of negative reactivity insertion within the system for each case and identify the most suitable option(s). The chosen configuration should be fast acting and capable of introducing a large amount of negative reactivity within the system even with a small gap. It should also provide a sufficient shutdown margin and various performance parameters will finally be governed by the exact regulatory requirements. For this study, it has been assumed that the system must have a shutdown worth of minimum 5000 pcm. This is sufficient at the current stage of design studies and should also satisfy the regulatory requirements in most countries [27]. It must be noted that the aim here is to identify the most suitable configuration and appropriate modifications can be made during the final design stages, if necessary.

**Fig 6:**
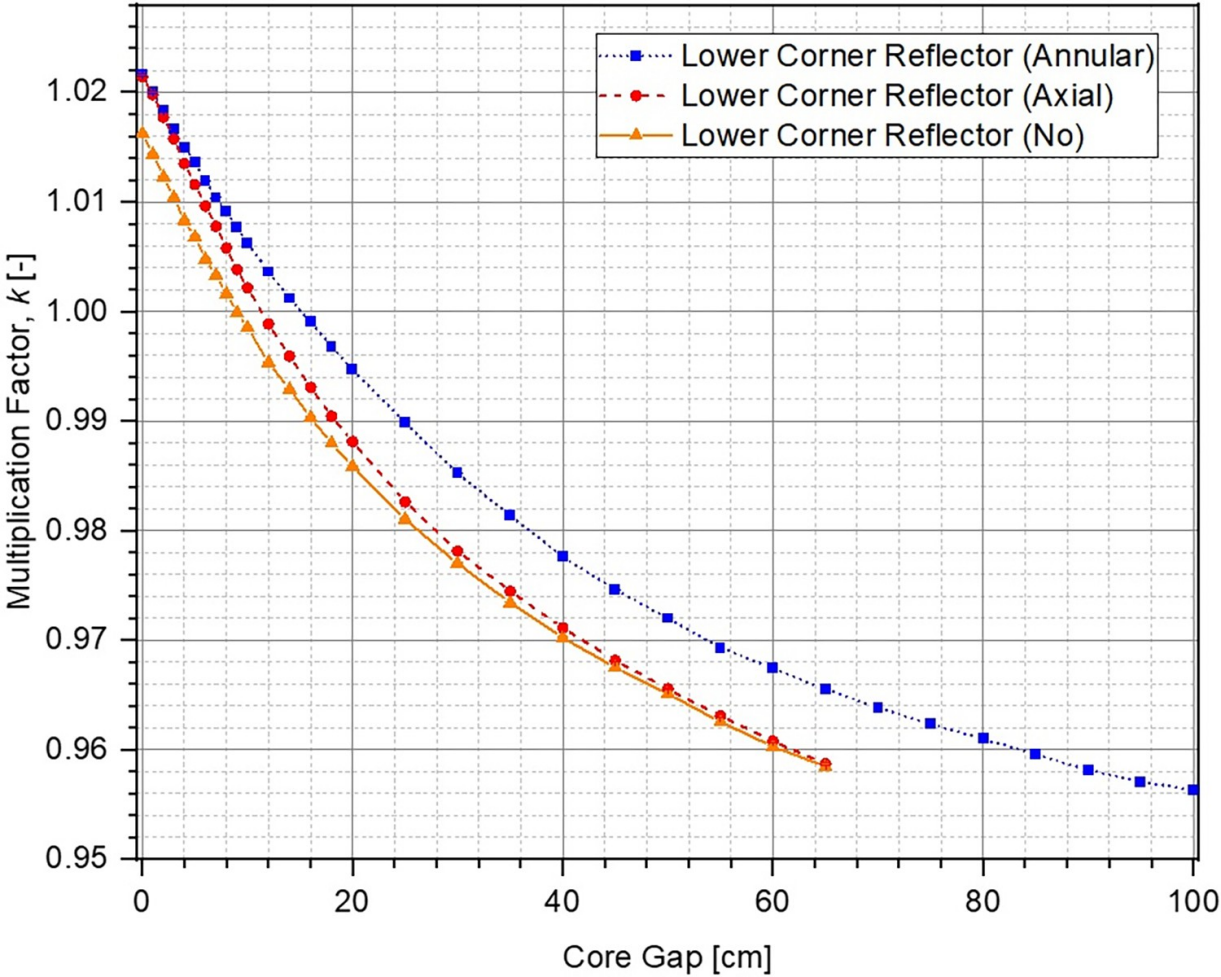
Multiplication factor at different core openings.

**Fig 7:**
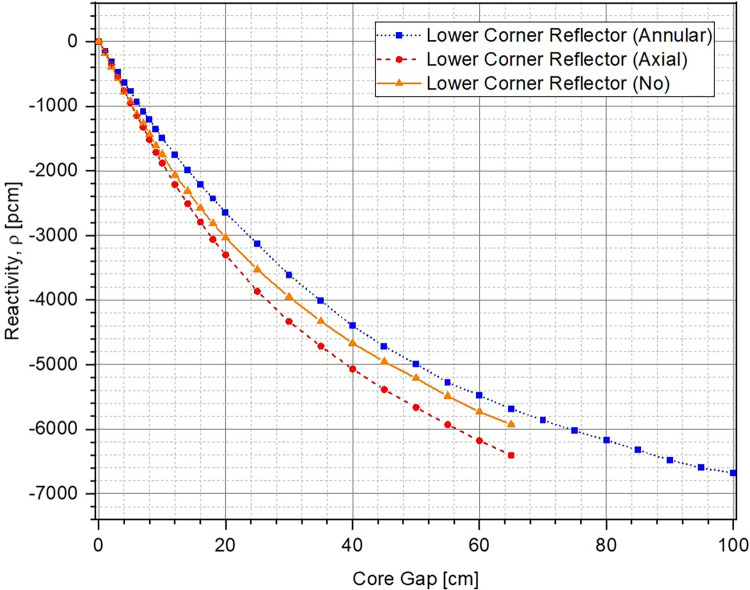
Reactivity insertion at different core openings.

The evolution in multiplication factor$\;\left(k\right)\;$of the system as a function of increasing core gap (opening of the lower core) from 0 cm to 100 cm has been shown in Fig 6. The corresponding reactivity insertion has been plotted in Fig 7. For each of the cases, multiplication factor reduces and higher negative reactivity$\;\left(\rho \right)\;$is inserted within the system as the lower core is moved further down. This is to be expected because the upper and the lower parts of the ZPE core become increasingly disassociated from each other with larger core gap which is equivalent to removal of fissile material from the system. If the core gap is increased further, k and ρ would ultimately reach asymptotic values indicating that the upper and lower parts of the ZPE are neutronically completely decoupled and exist as two independent smaller, subcritical systems. This has not been shown in Figs 6 and 7 for brevity. Importantly, there is sufficient shutdown margin within the system (> 6000 pcm) for all cases, which will allow required optimizations during the later stages.

**Fig 8:**
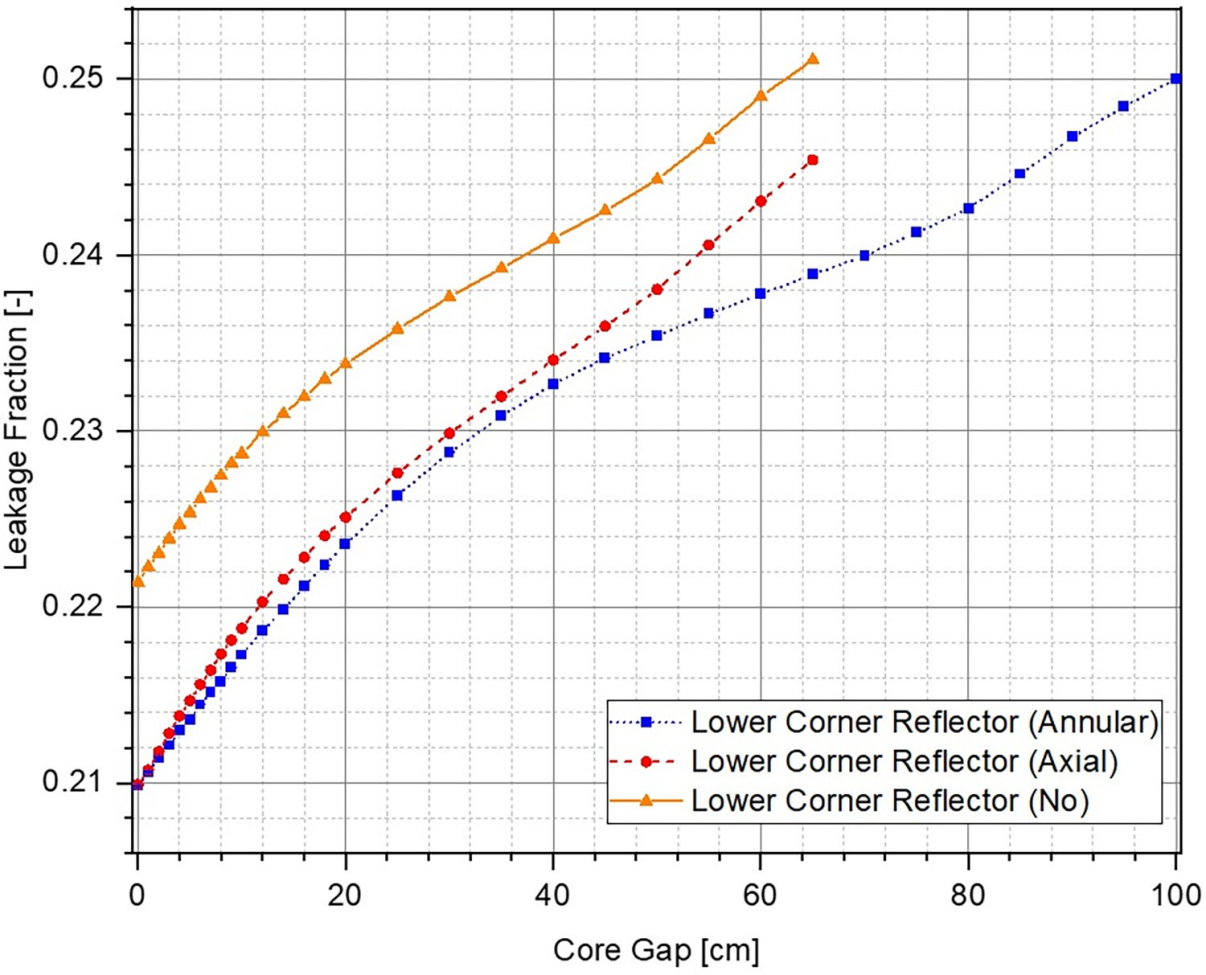
Leakage fraction at different core openings.

It can also be seen that in fully closed configuration (no core gap), the ZPE system with the lower corner reflector (dotted blue line with squares and dashed red line with circles) has a multiplication factor ~500 pcm higher than the case with no lower corner reflector (solid orange line with triangles). This is expected since the absence of the reflector would lead to higher neutron leakage, as can be seen from Fig 8, which shows the change in leakage fraction with increasing core gap. However, it is interesting to observe that the case with lower corner reflector as an extension of the lower annular reflector has a similar slope to that without the lower corner reflector. This shows that the rate of negative reactivity insertion (governed by the amount of neutron leakage) within the system with respect to core gap is similar, as can also be observed in Fig 7. The reactivity insertion in both cases is almost identical initially and differs by less than 500 pcm throughout the entire range of core gap studied here. The best performance (both, total worth and rate of reactivity insertion) is, however, seen for the case when lower corner reflector is present as a part of the lower axial reflector.

The reason for this interesting behavior in evolution of the multiplication factor and inserted reactivity with increasing core gap can be understood using the leakage fraction plot shown in Fig 8. In the fully closed configuration, neutron leakage is substantially lower in the presence of the lower corner reflector. Thus, the effect of every additional neutron leaking out of the system in these cases, as a result of separation between the lower and upper cores, is much higher than without the lower corner reflector. On the other hand, the differences seen between the cases with lower corner reflector are due to the different unreflected lower core area in the two cases. It is evident from Fig 5B and 5C (which have the same core gap) that the lower ZPE core has a significantly larger unreflected area when the lower corner reflector is a part of the lower axial reflector rather than the lower annular reflector. This results in higher neutron leakage in the former (red dashed curve) as compared to the latter (blue dotted curve).

Based on the analysis carried out in this section and the selection criteria highlighted earlier, the ZPE system with lower corner reflector as an extension of the lower axial reflector is the most viable and preferred option. However, the final design choice should be such that it does not interfere with the functioning of the control system and has been discussed in Section 3.3. Also, the final design choice must only be made after assessing the performance of the control system which has been investigated in Section 3.3.

### 3.3 Control system

**Fig 9:**
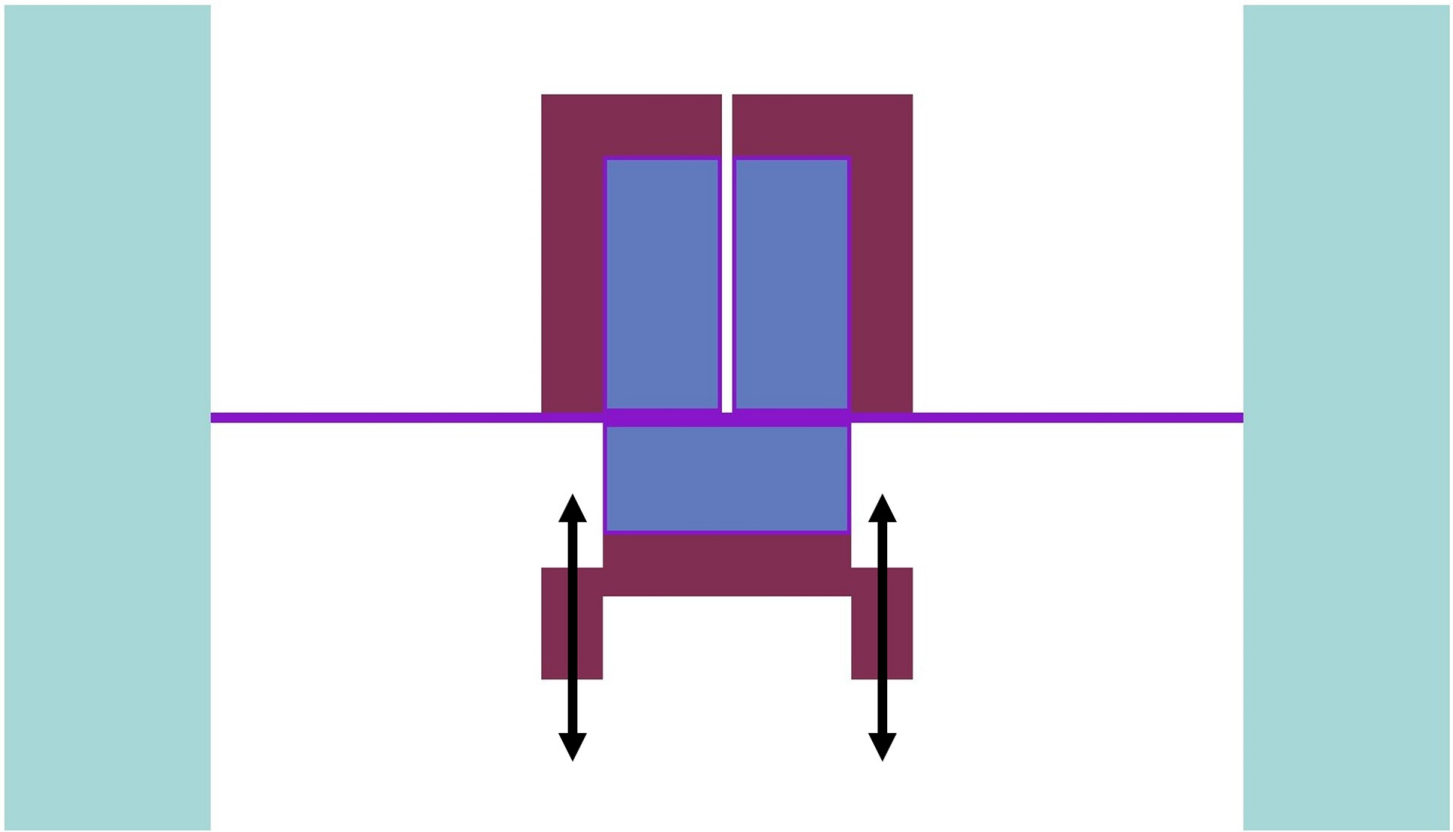
Control using annular reflector movement.

Design of the control system of the ZPE assembly is based on vertical movement of the annular reflector in the operational part, as shown in Fig 9. The idea is to control the reactor by manipulating neutron reflection, and thus leakage, within the system. This ensures the presence of diverse control and shutdown mechanisms for the ZPE system, which is an essential regulatory requirement. A detailed discussion on the control system design can be found elsewhere (see Ref. [6]). In principle, all cases described earlier in Section 3.2 are theoretically possible when evaluating the control options (not all have been illustrated here for brevity). However, the annular reflector will not be able to move freely if the lower corner reflector exists as an extension of the lower axial reflector. This could lead to a common cause failure and would violate the basic safety design principle of independent control and shutdown systems. Thus, only two different configurations will be possible:

1. ZPE without lower corner reflector
2. ZPE with lower corner reflector as an extension of the lower annular reflector

Similar to the analysis of shutdown systems in Section 3.2, both cases have been modeled using OpenMC with the calculation settings given in Section 2. The aim is to investigate the total worth of the control system as well as the rate of negative reactivity insertion in each case to identify the more suitable option. The chosen configuration should provide the necessary sensitivity while also providing sufficient control margin. This will be governed by the exact regulatory requirements as well as the sensitivity required for the experiments. It must be noted that the aim here is to identify the control configuration with better operational performance. The system can be modified appropriately during the final design stages, if necessary.

**Fig 10:**
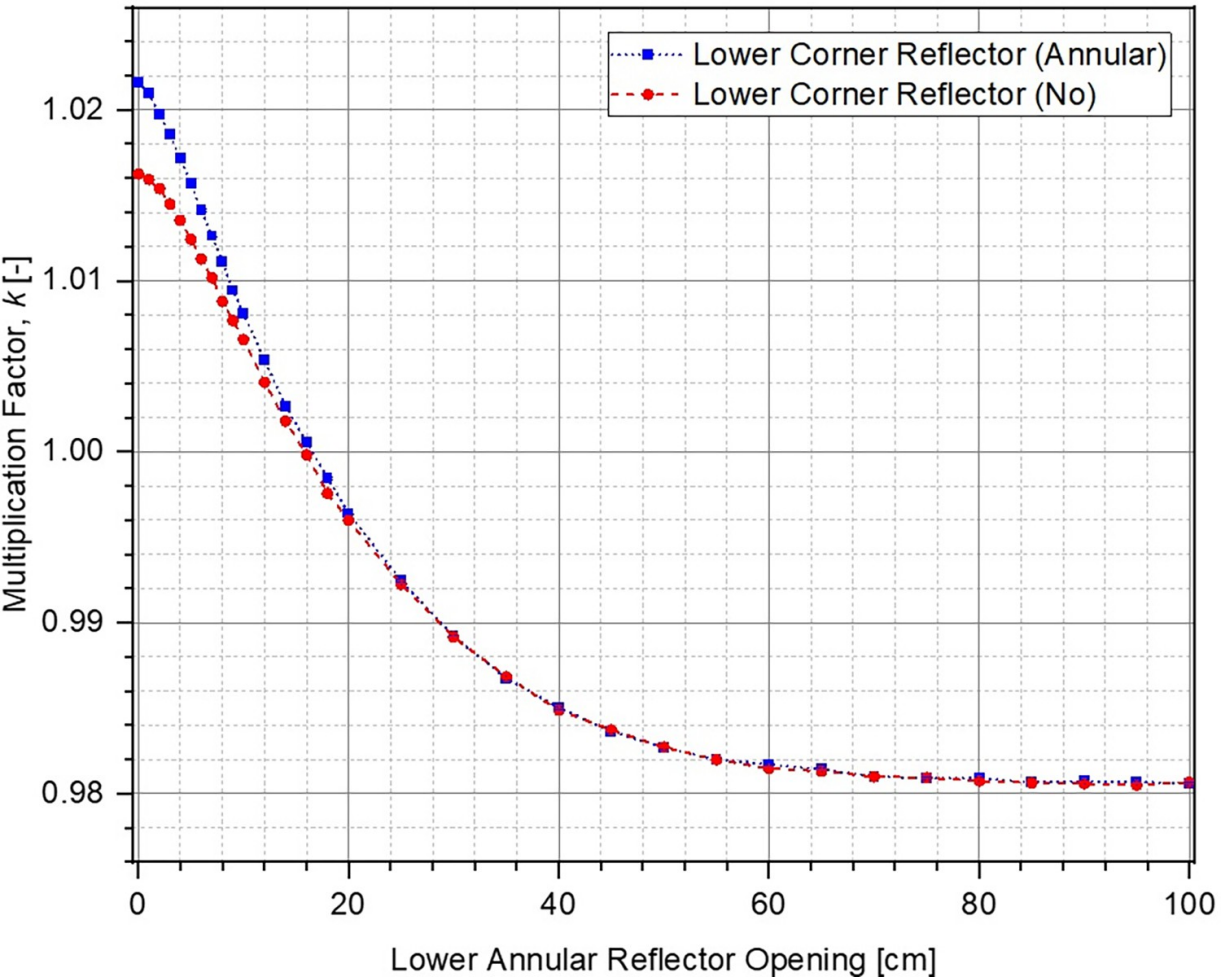
Multiplication factor at different lower annular reflector openings.

**Fig 11:**
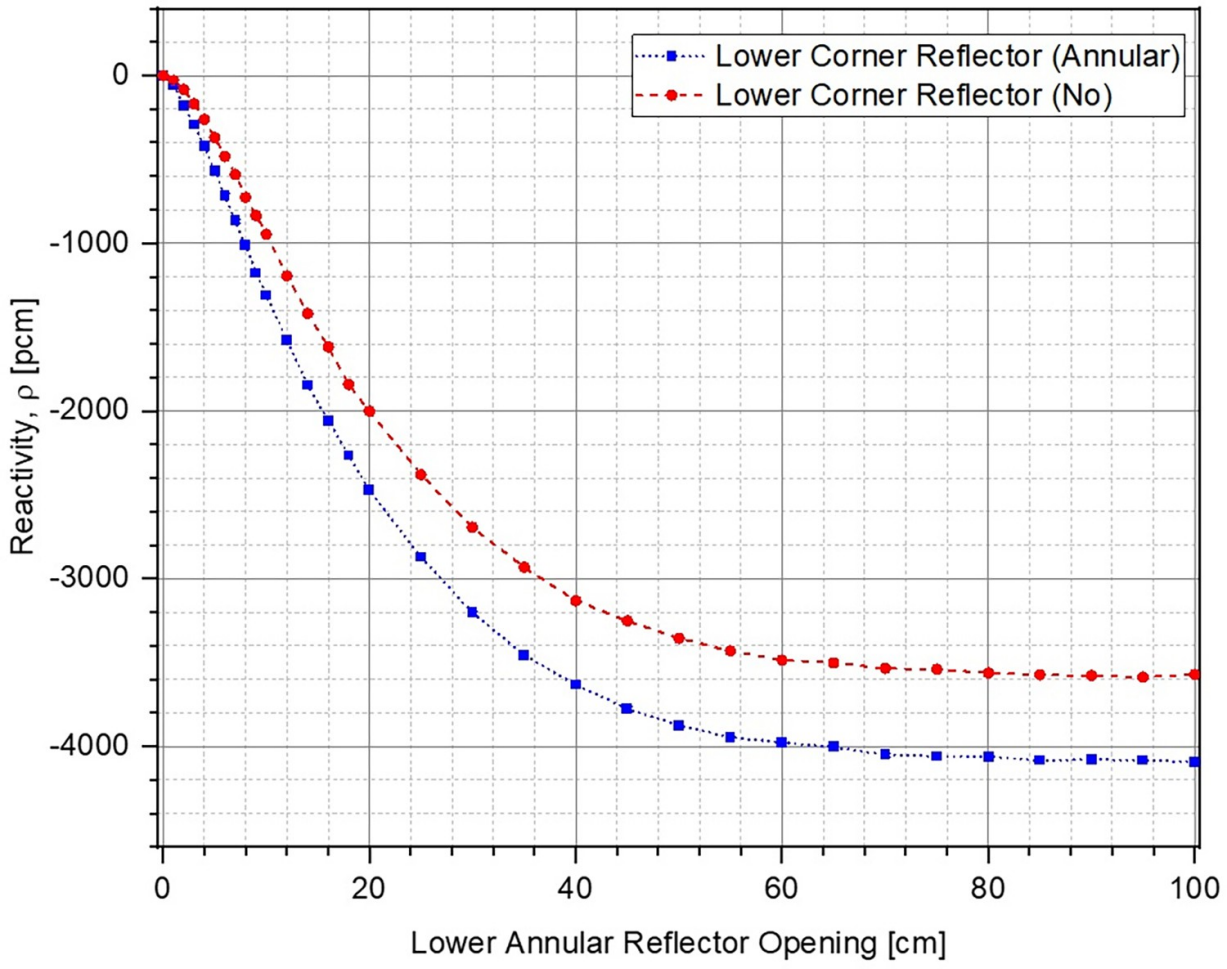
Reactivity insertion at different lower annular reflector openings.

Change in k of the system with respect to position of the lower annular reflector has been plotted in Fig 10. The reflector opening is varied from 0 cm (fully closed) to 100 cm. The corresponding reactivity inserted within the system has been shown in Fig 11. It can be observed that k decreases (higher negative reactivity insertion) as the lower reflector is moved further out before reaching asymptotic values at a reflector opening of ~60 cm. Increasing the reflector opening leads to increase in the unreflected area of lower part of the ZPE consequently allowing more neutrons to leak out of the system. As expected, for both cases, the ZPE assembly has the maximum criticality when the reflector is fully closed, thus, minimizing neutron leakage. Total worth of the control system for the case with (blue dotted curve with squares) and without (red dashed curve with circles) lower corner reflector are ~4100 pcm and ~3600 pcm, respectively. Even when considering the realistic operational range between 0 cm and 40 cm (where the control curves have a smooth behavior and haven’t saturated), the corresponding reactivity insertion is ~–3600 pcm and ~–3200 pcm, respectively. This should be sufficient for the typical experimental operations expected in a ZPE facility.

**Fig 12:**
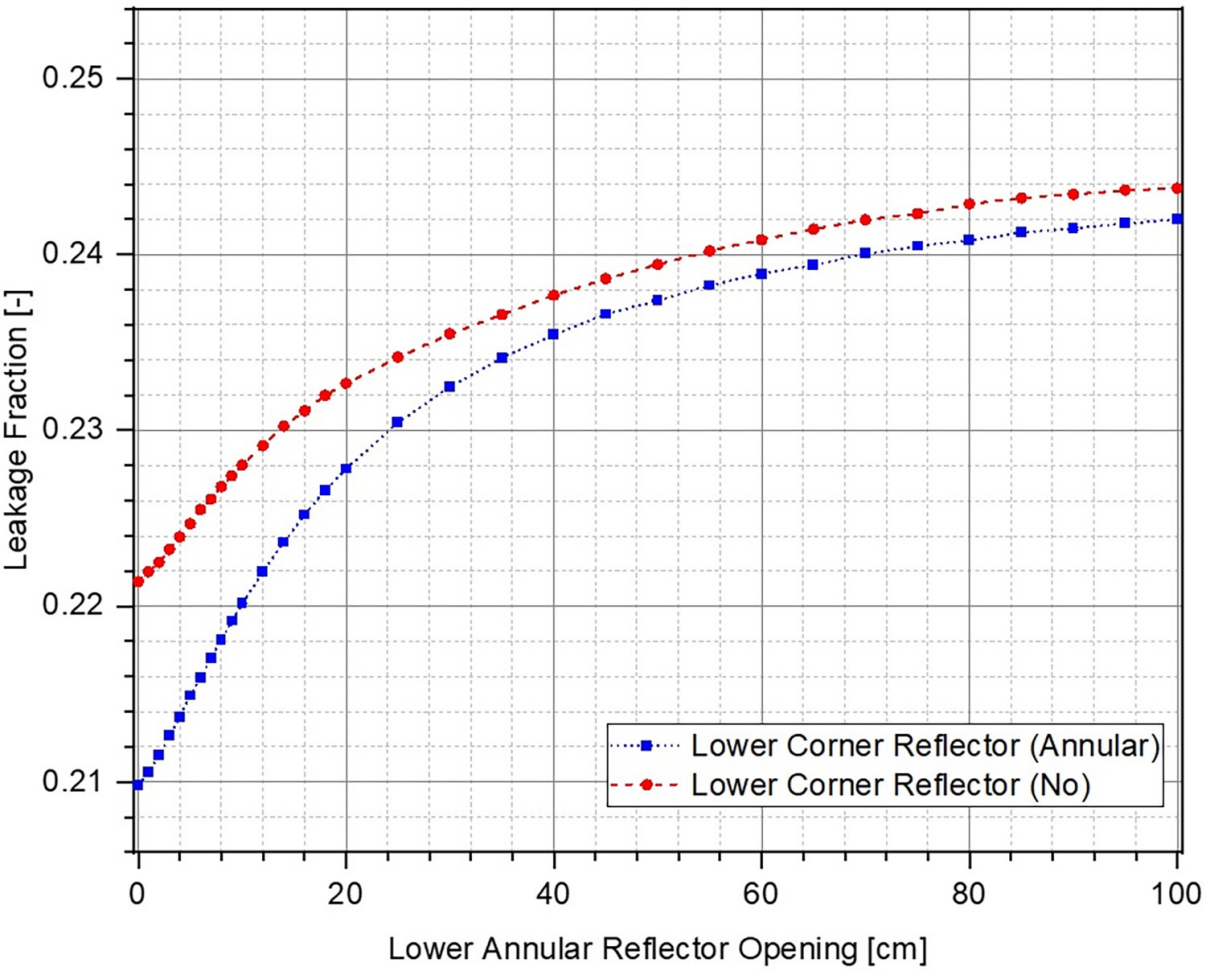
Leakage fraction at different lower annular reflector openings.

When comparing the two cases, it can be seen that the starting multiplication factor in the presence of lower corner reflector is higher than without it. This is expected as a higher fraction of neutrons can leak out of the system when the lower corner reflector is missing (see Fig 12). Due to this, the effect of every additional neutron leaking out of the system is higher in the presence of corner reflector, as also observed from the higher sensitivity of the control system (larger slope of the reactivity curve for the case with lower corner reflector) in Fig 11. Thus, it can be concluded that the case with lower corner reflector as an extension of the lower annular reflector offers a higher sensitivity as well as total control worth.

Using the results here along with discussions on the shutdown system presented in Section 3.2, it can be concluded that ZPE configuration with lower corner reflector as a part of the lower annular reflector is the best choice overall, when considering the operational or regulatory perspective. It provides sufficient worth, speed and sensitivity for both the shutdown and control systems, as well as satisfies basic safety principles. It must also be mentioned here that the ZPE system with lower corner reflector as an extension of the lower axial reflector, the most preferred option identified at the end of Section 3.2, was eliminated considering the engineering view as it constrained the movement of the control system.

### 3.4 Size of the experimental (upper) and operational (lower) parts

After analyzing the different configurations for their shutdown and control performance, and identifying the most suitable system, parametric studies have been carried out in this section to understand the operational envelope offered by considering different dimensions for the experimental and operational parts of the ZPE. Although, it might seem counter-intuitive to investigate the dimensions of the two parts here, this order is appropriate since the analyses performed earlier in Section 3.2 and Section 3.3 was aimed at understanding the different design options for shutdown and control systems and identifying the most preferable configuration. Optimizing the dimensions of the upper and lower parts of the ZPR will not fundamentally affect the behavior of the chosen shutdown and control mechanisms but will only change the available operational domain as well as experimental opportunities.

Ideally, the upper portion of the ZPE should be as large as possible for maximum homogeneity and minimizing any perturbations within experimental measurements arising from reactor operation (movement of the lower annular reflector for reactor control), heterogeneity within the system (material interfaces) or boundary effects. At the same time, it is essential that the lower part is large enough to fulfil the operational and regulatory requirements with adequate worth and sensitivity/speed for the shutdown and control systems. Finally, the combined system formed by the operational and experimental parts should have sufficient positive reactivity to allow ample operational maneuverability for different experiments. It should be noted here that the overall size of the system would also be constrained by economic as well as practical considerations, including the enrichment of U-235 in the fuel in the country which is taking action to build such a facility [6]. A total core height of 170 cm has been considered for the parametric studies reported in this section. The core radius has been fixed at 58 cm, as considered for the reference model leading to reasonable excess reactivity for experiments. The overall shutdown and control worth for different dimensions of the experimental and operational core have been shown in Table 7. Based on the results of this parametric study, an upper (lower) core height of 120 cm (50 cm), as considered in the reference model, is the most optimum choice. It adequately fulfils various operational as well as experimental constraints while balancing the different, often contrasting, requirements.

**Table 7:** Shutdown and control worth for various dimensions of the experimental (upper) and operational (lower) parts.

Core Height Upper/Lower [cm]	Shutdown Worth [pcm]	Control Worth [pcm]
140/30	4333.58 ± 10.67	2211.90 ± 10.55
130/40	5389.68 ± 11.49	3052.76 ± 10.87
120/50	6680.52 ± 10.50	4091.55 ± 11.70
110/60	8309.13 ± 11.21	5415.98 ± 10.78

### 3.5 Net shutdown and control worth under operational conditions

The analyses presented in Section 3.2 to Section 3.4 was focused on understanding the potential performance of different configurations for the shutdown and control systems, as well as the effect of different core heights for upper and lower parts of the ZPE. However, the initial criticality of the ZPE assembly was different in the presence and absence of lower corner reflector. In order to understand the total shutdown and control worth under operational conditions, further detailed investigations have been carried out in this section.

Firstly, a criticality search was performed for both cases, with and without the lower corner reflector, to determine the core radius for a starting criticality of 1.01 for the fully closed configuration. This would provide ~1000 pcm of excess reactivity within the system for operational maneuverability during experiments. A criticality search with these considerations led to a core radius of 56.5 cm and 57.2 cm, respectively, in the presence and absence of the lower corner reflector.

Next, the operational position of the control system was determined in each case (with and without lower corner reflector) using the corresponding core radius by performing another set of criticality searches. The operational position of the control system refers to the lower annular reflector opening for which the system has a multiplication factor of unity (1.0). The lower annular reflector opening was determined to be 7.5 cm and 10 cm, respectively, in the presence and absence of the lower corner reflector.

**Fig 13:**
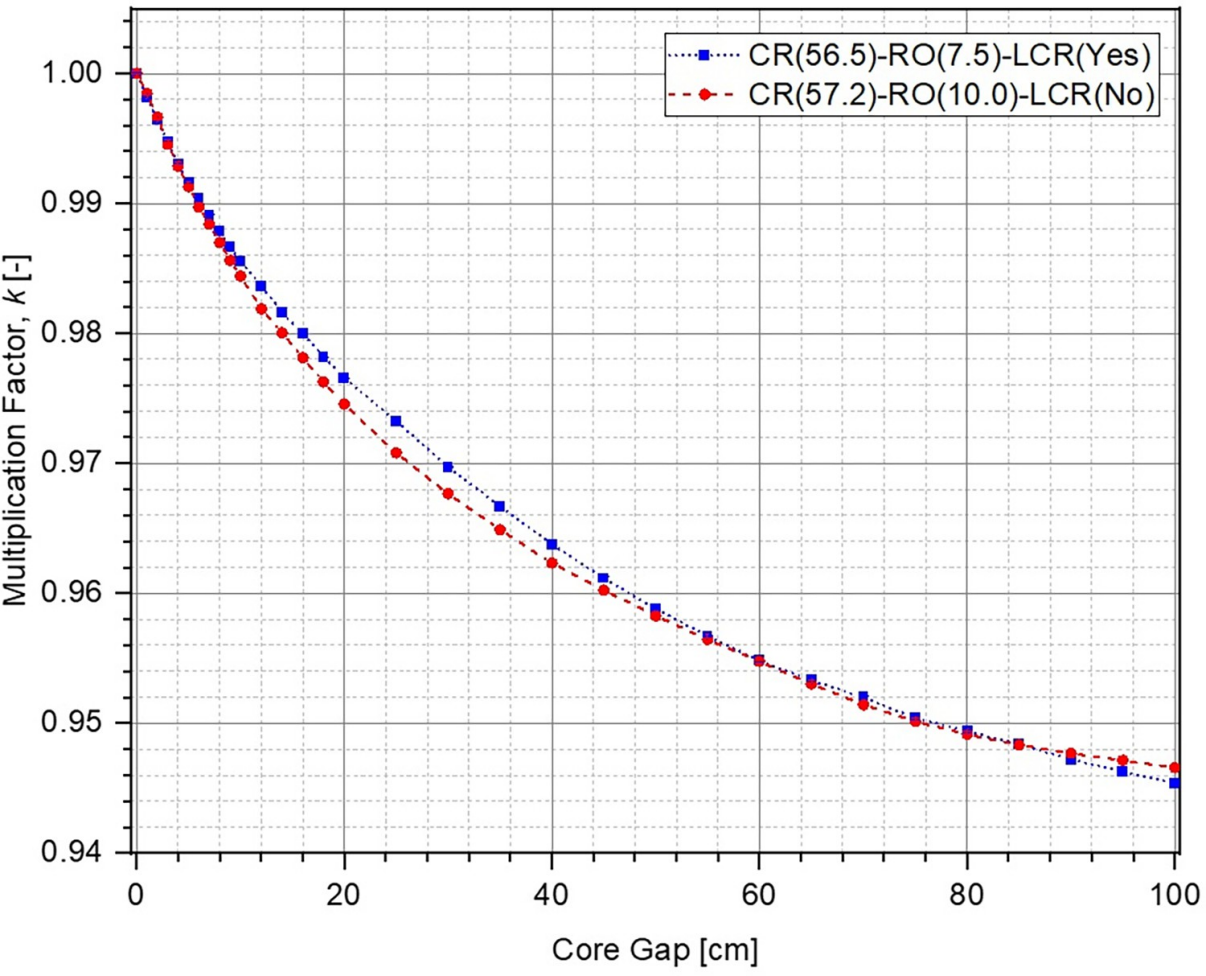
Multiplication factor at different core openings under operational conditions.

**Fig 14:**
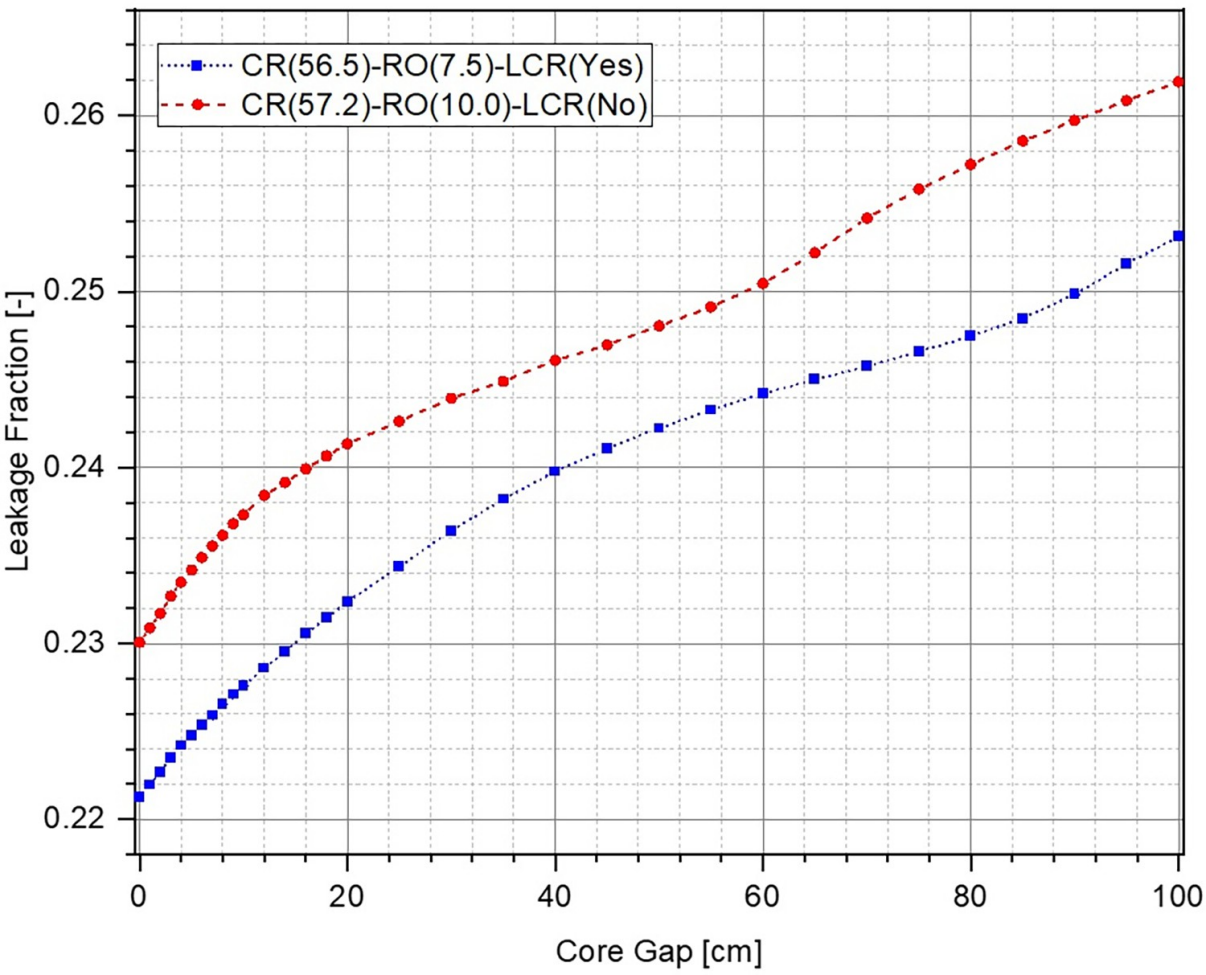
Leakage fraction at different core openings under operational conditions.

After determining the core radius and initial operational position of the lower annular reflector, performance of shutdown and control systems was investigated for each case. The core gap as well as lower reflector openings were varied between 0 cm and 100 cm, as done previously for the analyses presented in Section 3.2 and Section 3.3. The change in multiplication factor at different core openings for both cases has been shown in Fig 13. The corresponding leakage fractions have been plotted in Fig 14. The cases are represented using the following nomenclature:

CR$\left(core\;radius\right)$–RO$\left(reflector\;opening\right)$–LCR$\left(lower\;corner\;reflector\;present\right)$

**Fig 15:**
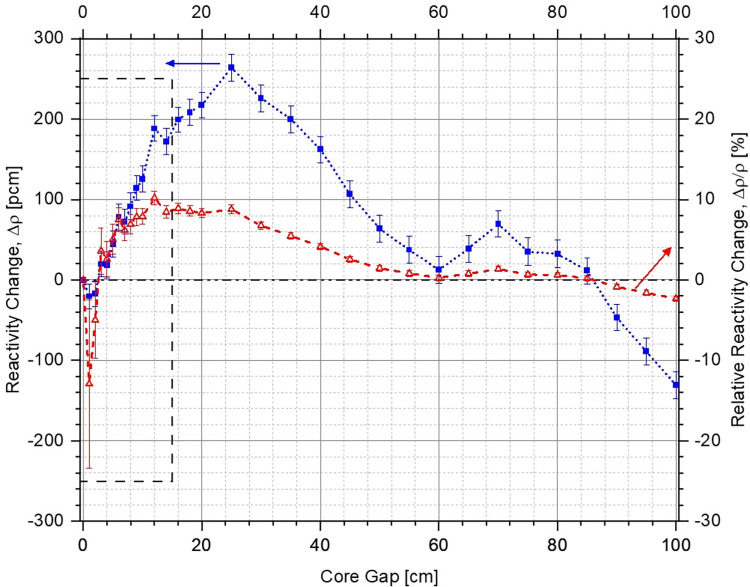
Change of reactivity inserted within the system with lower corner reflector in comparison to the case without the lower corner reflector as a function of core openings (positive and negative values indicate lower and higher negative reactivity insertion, respectively).

**Fig 16:**
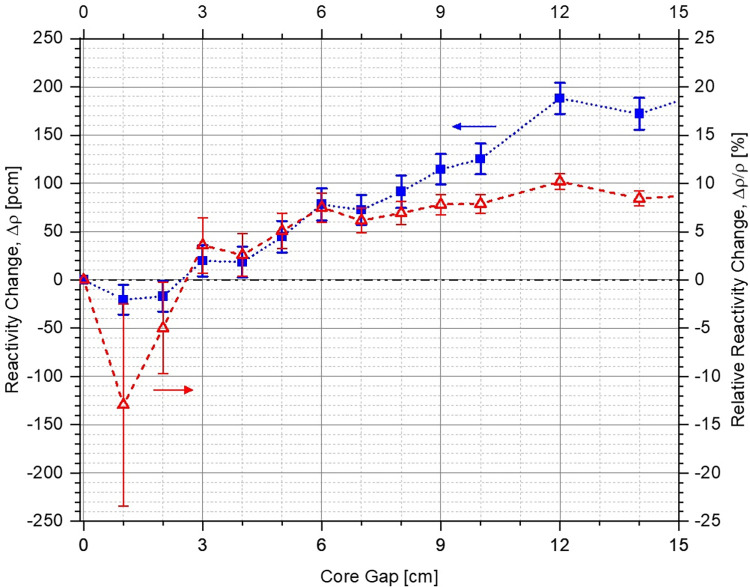
Magnified view of the change of reactivity inserted within the system with lower corner reflector in comparison to the case without the lower corner reflector as a function of core openings from 0 cm to 15 cm (positive and negative values indicate lower and higher negative reactivity insertion, respectively).

No significant difference is observed between the multiplication factor in the two cases for different core openings (see Fig 13). The negative reactivity insertion is almost identical for very small and large core openings, and the system without lower corner reflector (red dashed curve with circles) slightly outperforms the other case (blue dotted curve with squares) for intermediate core gaps. A maximum absolute difference of ~270 pcm (blue dotted curve with squares in Figs 15 and 16) is observed between the two cases for a core opening of 25 cm, as shown in Fig 15 (a magnified plot of the differences in reactivity inserted for core gaps of 0 cm to 15 cm has been shown in Fig 16). It is important to mention here that this is well within the error margin inherent in the design, the computational codes as well as the input cross-section data. In relative terms (red dashed curve with triangles Figs 15 and 16), the maximum difference in performance of the two configurations is ~10% at a core gap of 12 cm. This is marginal and the two cases can be considered to offer almost identical shutdown performance with a total shutdown worth of ~5500 pcm, which is greater than the chosen design criteria of > 5000 pcm. It must be noted here that the size of the system as well as operational lower annular reflector position are slightly different for the two configurations. This is also the reason for different leakage fractions for the two cases (see Fig 14) despite almost identical negative reactivity insertion. As mentioned earlier, the final design criteria would be fixed after exhaustive discussions with the regulators.

**Fig 17:**
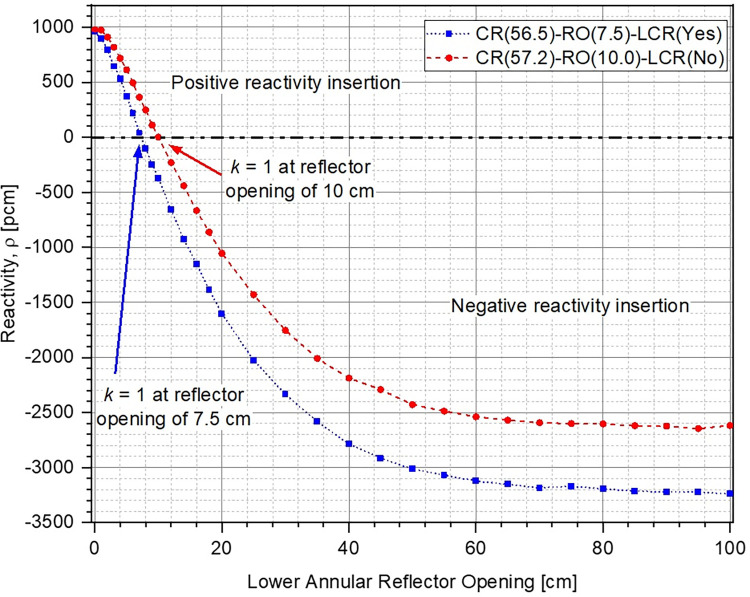
Reactivity (w.r.t. critical state, k = 1) at different lower annular reflector openings under operational conditions.

Finally, reactivity within the system (with respect to critical state, k = 1) for different lower annular reflector openings in each case has been plotted in Fig 17 following the same nomenclature, as used earlier for Figs 13 and 14. As mentioned previously, dimensions of the ZPE in each case (with and without lower corner reflector) have been chosen such that the system has a criticality of 1.01$\;\left(k\;=\;1.01\right)\;$in the fully closed configuration. This is equivalent to an excess reactivity of ~1000 pcm, as observed in Fig 17. The system attains a multiplication factor of unity$\;\left(k\;=\;1\right)\;$at reflector opening of 7.5 cm (blue dotted curve with squares) and 10 cm (red dashed curve circles), respectively, with and without the lower corner reflector. It can be seen that the control mechanism based on vertical movement of the lower annular reflector has an operational range of +1000 pcm to –3250 pcm with lower corner reflector. When the lower corner reflector is absent, the operational range is limited from +1000 pcm to –2600 pcm.

In conclusion, based on the results of shutdown and control performance presented in this section, it can be clearly seen that the ZPE configuration with the presence of lower corner reflector can offer more robust operation. The control system in this case has a larger total worth along with higher sensitivity while the shutdown performance is similar to the case without lower corner reflector.

## 4. ZPE system summary

This section summarizes the major design parameters of the ZPE system with lower corner reflector which has been chosen based on the analyses presented in Section 3. A novel split-core design has been proposed for the experimental ZPR in which the system is split into an upper (experimental) part and a lower (operational) part. The experimental portion of the ZPE is stationary with vertical and horizontal experimental channels while the lower core (along with the lower axial reflector) and the lower annular reflector can be moved vertically. Movement of the lower core and annular reflector serve as the shutdown and control systems, respectively. The two parts of the ZPR are separated by an intermediate stainless steel plate which also acts as the floor for the upper portion, thus eliminating the smallest possibility of inadvertent addition of positive reactivity within the system in the accidental scenario of the upper part falling onto the lower core due to a failure of the upper core anchoring during system shutdown. This innovative design also ensures complete independence of the reactor operation from all experiments.

The total core height of the ZPR has been chosen as 170 cm with upper and lower core heights of 120 cm and 50 cm, respectively, separated by a stainless steel plate of thickness 5 cm. The optimum core radius was found to be 56.5 cm and both portions of the core are contained in a 2 cm thick stainless steel vessel. The reactor core and vessel are surrounded in both the radial and axial directions by a 30 cm thick reflector of Hi-Si cast iron. Based on these parameters, the estimated total mass of the fuel salt and other materials has been summarized in Table 8.

**Table 8:** Estimated mass of different materials in the experimental Zero Power Reactor (ZPR) system.

Material	Mass [10^3^ kg]
Fuel salt	5.45
Stainless steel (vessel)	1.70
High silicon cast iron (reflector)	27.64
Stainless steel (floor)	9.93

**Fig 18:**
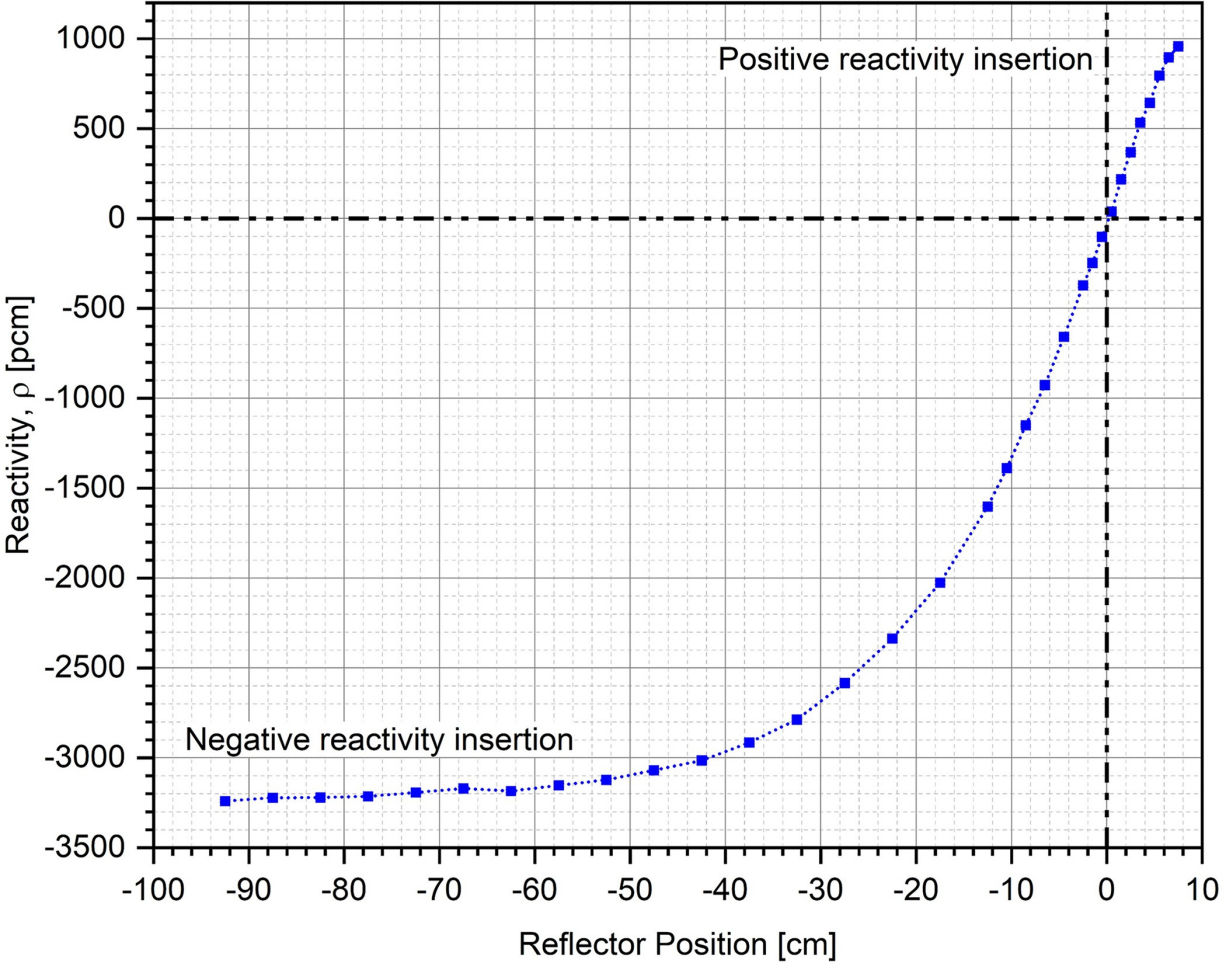
Control curve for the chosen ZPE system. Zero denotes the reference reflector position at which the system is critical$\;\left(k\;=\;1\right)\;$and corresponds to a reflector opening of 7.5 cm, as shown in Fig 18 (sign convention for reflector position–negative when the lower annular reflector opening is more than the reference position and positive otherwise).

The shutdown mechanism of the ZPR based on vertical movement of the lower core to modify the core gap offers a shutdown margin of more than 6000 pcm. The shutdown curve has been plotted earlier in Fig 7 (blue dotted curve with squares) and has not been reproduced here for brevity. Fig 18 shows the control curve for the chosen ZPE configuration, i.e., reactivity insertion in the system at different lower annular reflector positions. As mentioned earlier, control system based on annular reflector movement in the ZPR with lower corner reflector has a total control worth of around 4250 pcm from +1000 pcm to -3250 pcm. Thus, the control system provides sufficient operational maneuverability to perform various experiments by having ~1000 pcm of reserve positive reactivity. It can also insert large negative reactivity within the ZPR, offering a reasonable control margin.

## 5. Conclusions

With the increasing urgency of decarbonizing global energy systems while ensuring the availability and supply of reliable, 24/7 electricity for all, there is an ever-growing recognition of the importance of nuclear energy in the overall energy mix. In order to play this role, there is a strong need to develop novel nuclear technologies in addition to leveraging current reactor systems. A step-wise approach has previously been proposed for iMAGINE, an innovative technology based on molten salt reactors. A zero power experimental reactor is a crucial step in this approach for fast development with quick learning, active feedback and risk mitigation. The neutronic design of shutdown and control systems of the ZPE reactor based on iMAGINE has been presented in this paper.

Initially, a reference model for the ZPE has been presented along with a discussion of the Monte-Carlo results from different codes like OpenMC, MCNPX-2.7 and Keno-VI, including a parametric analysis to demonstrate how the initial dimensions for the reference model were obtained. This was followed by a description of the split-core design for the shutdown system. Advantages of this novel design with physically separated upper (experimental) and lower (operational) parts located in two different rooms were also presented. A shutdown worth in excess of 6000 pcm could be achieved for each configuration of the shutdown system that was considered. The highest total shutdown worth was observed in the case when the lower corner reflector was present as an extension of the lower axial reflector. However, this configuration could interfere with the proposed control system which uses vertical movement of the lower annular reflector and was thus, discarded. Next, performance of the control system was investigated in two configurations–with and without the lower corner reflector (as an extension of the lower annular reflector). While both configurations performed satisfactorily with a total control worth > 3600 pcm, it was seen that the control system had a higher total worth as well as sensitivity in the presence of lower corner reflector.

This was followed by parametric studies to understand the effect of different heights of the experimental and operational parts of the ZPE on its shutdown and control performance. It was found that heights of 120 cm and 50 cm for the upper and lower parts, respectively, were optimum considering the different constraints which included, sufficient control and shutdown performance while ensuring minimum perturbations in the experimental part. Finally, the net shutdown and control performances were investigated in a realistic operational scenario. After performing criticality searches to determine the core radius (for excess reactivity of ~1000 pcm in fully closed configuration for operational maneuverability during experiments) and lower reflector opening (control system position) for steady state operation, the total shutdown and control worth of the ZPE assembly in the presence and absence of lower corner reflector were calculated. The core radius was found to be 56.5 cm and 57.2 cm with and without the lower corner reflector, respectively. The corresponding lower reflector openings were 7.5 cm and 10 cm, respectively. It was also seen that while the shutdown performance was only marginally different in the two cases, performance of the control system was significantly better when the lower corner reflector was present. Thus, the system design with lower corner reflector as an extension of the lower annular reflector was chosen for ZPE reactor.

Finally, the system parameters of the chosen configuration including dimensions were summarized in Section 4. The total fuel salt inventory was estimated to be around 5.45 tons along with ~11.63 tons and ~27.64 tons of stainless steel and high-silicon cast iron, respectively. The shutdown system based on vertical movement of the lower core to modify the gap between the operational and experimental portions offered a large shutdown worth of ~6500 pcm. The control curve for the chosen configuration was also presented depicting the reactivity inserted within the system at different lower annular reflector positions. The control mechanism offered a robust operational envelope and allowed a reactivity insertion between +1000 pcm to -3250 pcm with sufficient speed and sensitivity.

In the future, detailed analysis will be performed to optimize the location of both vertical and horizontal instrumentation channels, as well as the detection and monitoring system. It would also be desirable to perform high fidelity simulations of potential experiments that would be of high interest for such a ZPE facility.

## References

[1] UN Department of Economic and Social Affairs, Sustainable Development. Ensure access to affordable, reliable, sustainable and modern energy for all [Internet]. Available from: https://sdgs.un.org/goals/goal7

[2] Government of United Kingdom. The Ten Point Plan for a Green Industrial Revolution. Dep Business, Energy Ind Strategy [Internet]. 2020;(November):1–38. Available from: https://www.gov.uk/government/publications/the-ten-point-plan-for-a-green-industrial-revolution

[3] Department for Business Energy and Industrial Strategy. Net Zero Strategy: Build Back Greener [Internet]. Gov.Uk. 2021. Available from: https://www.gov.uk/government/publications/net-zero-strategy

[4] Department for Energy Security and Net Zero. British nuclear revival to move towards energy independence [Internet]. 2023. Available from: https://www.gov.uk/government/news/british-nuclear-revival-to-move-towards-energy-independence

[5] Birol F. Nuclear Power in a Clean Energy System [Internet]. 2019. Available from: https://www.iea.org/reports/nuclear-power-in-a-clean-energy-system

[6] Merk B, Noori-kalkhoran O, Jain L, Aflyatunova D, Jones A, Powell L, et al. A Draft Design of a Zero-Power Experiment for Molten Salt Fast Reactor Studies. Energies. 2024;1–15. https://doi.org/10.3390/ en17112678 10.1371/journal.pone.0309928 PMC11482688 39413066

[7] Department Of Energy. At COP28, Countries Launch Declaration to Triple Nuclear Energy Capacity by 2050, Recognizing the Key Role of Nuclear Energy in Reaching Net Zero [Internet]. 2023. Available from: https://www.energy.gov/articles/cop28-countries-launch-declaration-triple-nuclear-energy-capacity-2050-recognizing-key

[8] World Nuclear Association. Generation IV Nuclear Reactors [Internet]. 2024. Available from: https://world-nuclear.org/information-library/nuclear-fuel-cycle/nuclear-power-reactors/generation-iv-nuclear-reactors

[9] NS-Energy. Profiling the top nuclear power pros and cons [Internet]. 2021. Available from: https://www.nsenergybusiness.com/features/newstop-nuclear-power-pros-and-cons-5760814/

[10] Merk B, Litskevich D, Peakman A, Bankhead M. IMAGINE—A Disruptive Change to Nuclear or How Can We Make More Out of the Existing Spent Nuclear Fuel and What Has to be Done to Make it Possible in the UK? atw-Internationale Zeitschrift fuer Kernenergie. 2019;64(6–7).

[11] Merk B, Litskevich D, Detkina A, Noori-kalkhoran O, Jain L, Derrer-Merk E, et al. iMAGINE—Visions, Missions, and Steps for Successfully Delivering the Nuclear System of the 21st Century. Energies. 2023; doi: 10.3390/en16073120

[12] Jain L, Merk B, Derrer-Merk E. iMAGINE– 7,500 YEARS OF SUSTAINABLE ENERGY FROM NUCLEAR WASTE. Sci Parliment [Internet]. 2023;79(3):10–2. Available from: https://www.scienceinparliament.org.uk/wp-content/uploads/2024/05/iMAGINE-–-7500-years-of-sustainable-energy-from-nuclear-waste.pdf

[13] Rosatom. A workshop on the development of a liquid-salt reactor was held at the MCC [Internet]. Available from: https://sibghk.ru/news/9068-na-gkhk-proshlo-rabochee-soveshchanie-po-voprosu-sozdaniyazhidkosolevogo-reaktora.html?_x_tr_sl=ru&_x_tr_tl=en&_x_tr_hl=en-GB

[14] Sowder A. Program on Technology Innovation: Government and Industry Roles in the Research, Development, Demonstration, and Deployment of Commercial Nuclear Reactors: Historical Review and Analysis. 2017;134.

[15] Groves LR. Now It Can Be Told: The Story Of The Manhattan Project. New York; 1962. 491 p.

[16] NEA. The demise of zero power reactors: Addressing reduced experimental capabilities [Internet]. 2023. Available from: https://www.oecd-nea.org/jcms/pl_83239/the-demise-of-zero-power-reactors-addressing-reduced-experimental-capabilities

[17] Merk B, Detkina A, Atkinson S, Litskevich D, Cartland-Glover G. Evaluation of the breeding performance of a NaCl-UCl-Based reactor system. Energies. 2019;12(20). 10.3390/en12203853

[18] Dordevic V, Gulisija Z, Mihailovic M, Acimovic-pavlovic Z, Antic M. Corosion Resistant High Silicon Cast Irons Characteristics of Corrosion Resistant High Silicon. Proc 3rd BMC-2003-Ohrid, R Maced. 2003;333–7.

[19] PNNL. Data Mining Analysis and Modeling Cell: Compendium of Material Composition Data for Radiation Transport Modeling. 2021;(April):1–298. Available from: www.pnnl.gov/main/publications/external/technical_reports/pnnl-15870rev1.pdf

[20] Romano PK, Horelik NE, Herman BR, Nelson AG, Forget B, Smith K. OpenMC: A state-of-the-art Monte Carlo code for research and development. Ann Nucl Energy [Internet]. 2015;82:90–7. Available from: 10.1016/j.anucene.2014.07.048

[21] Chadwick MB et al. ENDF/B-VII.1 nuclear data for science and technology: Cross sections, covariances, fission product yields and decay data. Nucl Data Sheets. 2011;112(12):2887–996. doi: 10.1016/j.nds.2011.11.002

[22] Pelowitz DB. Mcnpx User ‘ S Manual- Version 2.7.0. 2011.

[23] Petrie LM, Bekar KB, Celik C. SCALE 6.2.3 User Manual. ORNL/TM-2005/39. Oak Ridge; 2018.

[24] Rearden BT, Jessee MA. SCALE Code System Version 6.2.3—ORNL/TM-2005/39 [Internet]. 2018. 1–2760 p. Available from: 10.2172/1426571

[25] Studvik. HELIOS2 METHODS (version 2.03.01) SSP-11/452 Rev 6. 2021.

[26] Noori-kalkhoran O, Litskevich D, Detkina A, Jain L, Cartland-glover G, Merk B. On the Employment of a Chloride or Fluoride Salt Fuel System Properties and Core Criticality. Energies. 2022;15:1–20. 10.3390/en15238865

[27] IAEA, Safety margins of operating reactors. Int Atomic Energy Agency. 2003;(January):143.

